# Emperipolesis mediated by CD8^+^ T cells correlates with biliary epithelia cell injury in primary biliary cholangitis

**DOI:** 10.1111/jcmm.14752

**Published:** 2019-12-18

**Authors:** Su‐xian Zhao, Wen‐cong Li, Na Fu, Guang‐de Zhou, Shu‐hong Liu, Li‐na Jiang, Yu‐guo Zhang, Rong‐qi Wang, Yue‐min Nan, Jing‐min Zhao

**Affiliations:** ^1^ Department of Traditional and Western Medical Hepatology The Third Hospital of Hebei Medical University Shijiazhuang P.R. China; ^2^ Department of Pathology and Hepatology Institution The Fifth Medical Center, General Hospital of PLA Beijing P.R. China

**Keywords:** apoptosis, biliary epithelial cells, CD8^+^ T cell, Emperipolesis, primary biliary cholangitis

## Abstract

Primary biliary cholangitis (PBC) is an autoimmune disease characterized by chronic destruction of the bile ducts. A major unanswered question regarding the pathogenesis of PBC is the precise mechanisms of small bile duct injury. Emperipolesis is one of cell‐in‐cell structures that is a potential histological hallmark associated with chronic hepatitis B. This study aimed to clarify the pathogenesis and characteristics of emperipolesis in PBC liver injury. Sixty‐six PBC patients, diagnosed by liver biopsy combined with laboratory test, were divided into early‐stage PBC (stages I and II, n = 39) and late‐stage PBC (stages III and IV, n = 27). Emperipolesis was measured in liver sections stained with haematoxylin‐eosin. The expressions of CK19, CD3, CD4, CD8, CD20, Ki67 and apoptosis of BECs were evaluated by immunohistochemistry or immunofluorescence double labelling. Emperipolesis was observed in 62.1% of patients with PBC, and BECs were predominantly host cells. The number of infiltrating CD3^+^ and CD8^+^ T cells correlated with the advancement of emperipolesis (*R* ^2^ = 0.318, *P* < .001; *R* ^2^ = 0.060, *P* < .05). The cell numbers of TUNEL‐positive BECs and double staining for CK19 and Ki67 showed a significant positive correlation with emperipolesis degree (*R* ^2^ = 0.236, *P* < .001; *R* ^2^ = 0.267, *P* < .001). We conclude that emperipolesis mediated by CD8^+^ T cells appears to be relevant to apoptosis of BEC and thus may aggravate the further injury of interlobular bile ducts.

## 1 INTRODUCTION

Primary biliary cholangitis (PBC) is an autoimmune disease characterized by chronic destruction of the bile ducts resulting in cholestasis, portal inflammation and fibrosis that ultimately leads to progressive ductopenia and cirrhosis.^1^ Orthotopic liver transplantation is to date the only definitive treatment approach for end‐stage PBC.

A major unanswered question regarding the pathogenesis of PBC is the precise mechanisms of small duct injury and destruction. Although several findings suggest that biliary epithelial cell (BEC) apoptosis may be of considerable importance for understanding PBC,^2^, ^3^, ^4^ the exact mechanism of cellular destruction remains to be elucidated. On histological sections, lymphocytes are commonly detected in the close vicinity of the damaged small bile ducts.

Emperipolesis is one of cell‐in‐cell structures that have been observed in chronic viral hepatitis.^5^ Recently, a study on auto‐immune hepatitis showed emperipolesis to be predominantly mediated by CD8^+^ T cells, which appeared to induce apoptosis of hepatocytes and thus revealing another potential mechanism of autoimmune hepatocyte injury.^6^ In our previous study, we had shown that emperipolesis was frequently seen in PBC, while BEC was predominantly host cells.^7^ However, it remains unknown whether emperipolesis is involved in BEC injury. Our present study aims to define the role of emperipolesis in bile duct injury in PBC and to identify the cell types entering BEC concerning the chronic nonsuppurative destructive cholangitis by analysing the infiltrating cells around the damaged bile duct in PBC patient samples.

## 2 MATERIALS AND METHODS

### 2.1 Subjects

Liver biopsy specimens were obtained from all patients histologically characterized as early PBC (E‐PBC, stages I and II, n = 39) and late PBC (L‐PBC, stages III and IV, n = 27) at the Third Hospital of Hebei Medical University and the Fifth Medical Center, General Hospital of PLA (Table 1). These patients, whose average ages were 43.5 ± 7.1 years, included 59 women and 7 men. With approval from our institutional review board, all subjects provided written informed consent prior to enrollment in the study. The diagnosis was made by serum biochemistry and clinical features according to EASL and AASLD guidance, and all subjects were double‐checked by liver biopsy.^1^, ^8^ Liver specimens fulfilled the needle biopsy standard (1.5 cm length, portal tract number no <8), and the evaluations were undergone blinded. All other infections or liver diseases were excluded from the selected type of disease. All patients were provided informed consent according to a protocol approved by the Fifth Medical Center, General Hospital of PLA and Hebei Medical University. Demographic and biochemistry data of all cases were shown in Table 1.

**Table 1:** Demographic characteristics and laboratory parameters of patients with PBC and controls

Disease	E‐PBC（n = 39）	L‐PBC（n = 27）	*P* value
Age(y)	37.2 ± 2.7	46.9 ± 4.1	<.05
Female/Male	34/5	24/3	<.05
ALT (U/L)	293.0 ± 100.1	118.4 ± 45.2	<.05
AST (U/L)	163.2 ± 52.8	131.7 ± 43.1	NS
TBil (μmol/L)	38.6 ± 14.2	92.1 ± 38.2	<.05
DBil (μmol/L)	24.1 ± 8.3	67.3 ± 23.6	<.05
ALP (U/L)	419.0 ± 142.0	467.0 ± 91.2	NS
γ‐GT (U/L)	533.0 ± 180.6	447.0 ± 152.3	NS

Note

Continuous variables are expressed as mean ± SD; NS, not significant differences.

### 2.2 Clinical and laboratory assessments

Clinical examination and conventional liver function tests were assessed in all patients. Serum biochemical and virological parameters were detected in patients. Serum aspartate aminotransferase (AST), alanine aminotransferase (ALT), alkaline phosphatase (ALP), γ‐glutamyltranspeptadase (GGT), total bilirubin (TBil), hepatitis B surface antigen (HBsAg), hepatitis B e‐antigen (HBeAg), hepatitis B core antigen (HBcAg), antibody to hepatitis B surface antigen (HBsAb) and antibody to hepatitis B e‐antigen (HBeAb) were detected in clinical laboratory. Serum antinuclear antibodies (ANA), smooth muscle antibodies (SMA) and antimitochondrial antibodies (AMA) were determined using indirect immunofluorescence (IIF) on Hep‐2 cell, monkey liver, rat kidney and rat stomach tissue slides. Serum antibodies against liver‐kidney microsomes type 1 (LKM‐1) and soluble liver antigen/liver‐pancreas antigen (SLA/LP) were assessed using immunoblotting assays (Euroassay test kit, Euroimm ‐un, Lubeck, Germany).

### 2.3 Antibodies

Antibodies used were as follows: anti‐CD3 (1:80 for Immunohistochemistry (IHC), Abcam), anti‐CD4 (1:100 for IHC, Zymed Laboratories, Santiago, USA), anti‐CD8 (1:200 for Immunofluorescence (IF) and IHC, Abcam), anti‐CD20 (1:200 for IF and IHC, Abcam), anti‐ki67 (1:100 for IHC, Zymed Laboratories, Santiago, USA) and anti‐CK19 (1:50 for IHC, Zymed Laboratories, Santiago, USA). Alexa 594 goat antimouse (1:200 for IF, Abcam), Alexa 488 goat anti‐rabbit (1:200 for IF, Abcam), DAPI (Southern Biotech) were used in IF. Ultra View Universal Alkaline Phosphatase Red Detection Kit (VENTANA), Ultra View Universal DAB Detection Kit (VENTANA) and Vector SK‐5300 AP Substrate Kit, Vector SK‐5400 BCIP/NBT (Zymed Laboratories) were used for IHC double staining.

### 2.4 Immunohistochemistry

Rabbit polyclonal antibodies against human CD3, CD4, CD8 and CD20 were used for immunohistochemical staining of liver. Tissue sections were cut by 4 μm from paraffin‐embedded tissue blocks and placed on slides. After deparaffinization, slides were exposed to microwave pretreatment in 10 mmol/L sodium citrate buffer (pH 6.0) at 98°C for 15 minutes for antigen retrieval. Endogenous peroxidase activity was blocked by incubation in 3% hydrogen peroxide for 20 minutes. Sections were incubated with the primary antibody of CD3, CD4, CD8 and CD20. After washed with phosphate‐buffered saline (PBS), sections were incubated with Envision system (Dako, Glostrup, Denmark) at room temperature for 1 hour. After benzidine reaction, sections were counterstained with haematoxylin. Negative controls were run by replacing the primary antibody with PBS.

Semi‐quantitative analysis was performed for immunoreactivity. Five representative hepatic lobules and portal tracts were randomly selected in each section. The populations of CD3, CD4, CD8 and CD20 positive cells were assessed by counting five random fields (magnification, ×200) using a standard grid. Values are given as the mean ± standard deviation (SD).

### 2.5 IHC double staining and IF staining

Samples were pretreated suitably for each antibody, respectively. IHC double staining was made according to Instruction Manual. IF staining was performed to show the infiltrating cells for distinguishing individual cell types as follows: primary antibodies (Anti‐cholangiocyte and Anti‐CD8, Anti‐cholangiocyte and Anti‐CD20, Anti‐cholangiocyte and Anti‐ki67) which were incubated at 4°C overnight. Slides were washed 3 times with PBS for 5 minutes. Different secondary antibodies were separately incubated for 30 minutes at 37°C. DAPI was used to stain the nucleus in IF. IHC double staining was performed following the instructions of IHC double staining kit. Confocal microscopy was used to visualize the histological IF staining. A Carl Zeiss LSM 710 laser scanning system and LAM Image Browser software (Olympus, USA) were used to capture images.

### 2.6 TUNEL apoptosis detection

The sections were deparaffinized with routine process. The specimens were then rinsed in PBS for 5 minutes and incubated with proteinase K (20 μm/mL) for 15 minutes at 37°C. The slides were then rinsed in PBS 3 times for 5 minutes each. DNA fragmentation was detected using TUNEL Apoptosis Detection Kit (Merck Millipore, German). DAPI was used to stain the nucleus.

### 2.7 Liver histopathological assessment

Liver biopsies, performed using a 16‐gauge needle, were fixed in 10% neutral buffered formalin and embedded in paraffin before 4‐μm‐thick sections were cut. Sections were stained with haematoxylin and eosin (H&E) and Masson methods. All liver tissue specimens were independently reviewed blindly by two liver pathologists. Emperipolesis was evaluated on H&E stained slides. To reduce sampling error, the frequency of emperipolesis per portal tract was determined. The cellular infiltration numbers of CD3^+^, CD4^+^, CD8^+^ and CD20^+^ were scored in immunohistochemically stained liver sections with each portal area visualized using a 200× objective. All samples were diagnosed by 3 hepato‐pathologists blindly. Emperipolesis was evaluated on both H&E stained, double‐colour IHC and IF stained slides.

### 2.8 Statistical analysis

SPSS 17.0 statistical software (SPSS Inc) was used to perform statistical analysis. Analysis of variance (ANOVA) test, least significant difference (LSD) test, Pearson`s Chi‐square (*χ* ^2^) test and Student's *t* test were performed to evaluate quantitative variables. The results were considered statistically significant, as the *P* value was <.05. The pathological diagnoses were triply verified by 3 hepato‐pathologists.

## 3 RESULTS

### 3.1 The clinical characteristics of PBC patients

Totally, 66 patients were enrolled in our study, which were divided into E‐PBC group (n = 39) and L‐PBC group (n = 27). In E‐PBC group, there are 34 females and 5 males, while in L‐PBC group, females and males were 24 and 3, respectively. Compared with E‐PBC group, the patients suffering L‐PBC were much elder (46.9 ± 4.1 vs. 37.2 ± 2.7 years). By collecting the liver function data, we found that patients in E‐PBC group had higher levels in ALT (293.0 ± 100.1 vs. 118.4 ± 45.2 U/L), AST (163.2 ± 52.8 vs. 131.7 ± 43.1 U/L), γ‐GT (533.0 ± 180.6 vs. 447.0 ± 152.3 U/L), and lower levels in TBil (38.6 ± 14.2 vs. 92.1 ± 38.2 μmol/L), DBil (24.1 ± 8.3 vs. 67.3 ± 23.6 μmol/L), ALP (419.0 ± 142.0 vs. 467.0 ± 91.2 U/L) than L‐PBC group. However, there were no significance in the levels of AST, ALP, γ‐GT between the two groups (Table 1).

### 3.2 Histological features of liver tissues from PBC patients and emperipolesis

**Figure 1:**
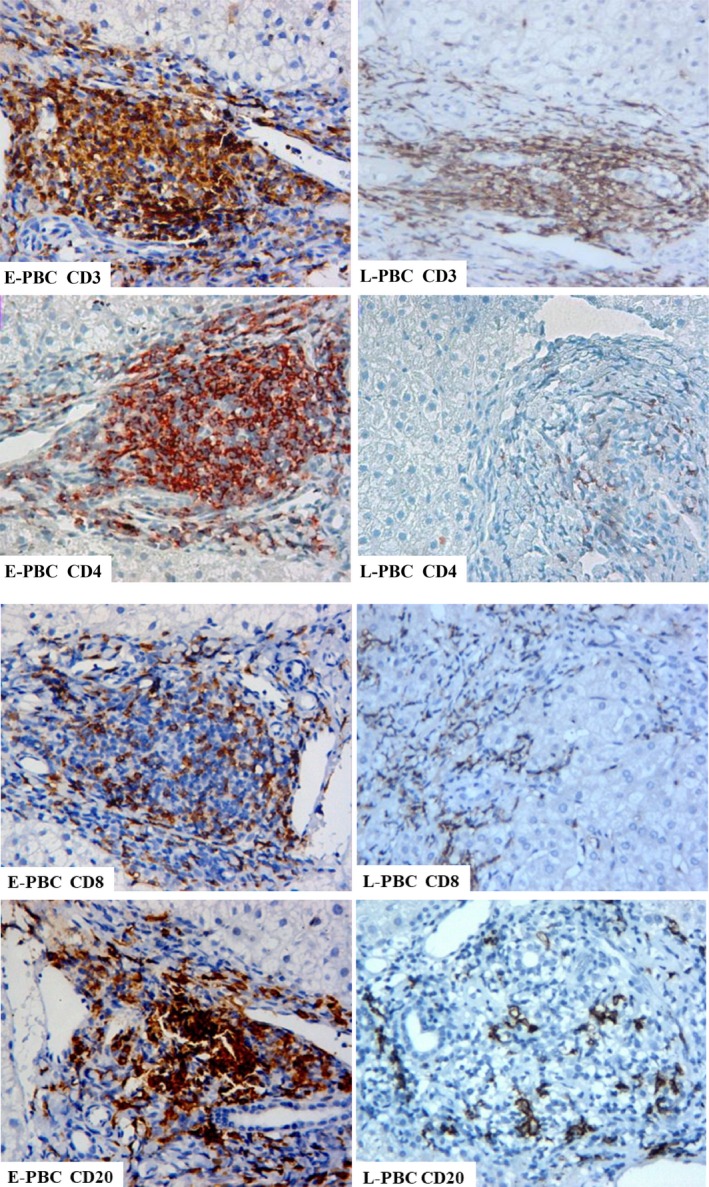
Types of infiltrating immune cells in the portal area from patients with PBC. Representative images of CD3^+^, CD4^+^and CD8^+^ T cell and B cell (CD20) staining of the portal area from liver biopsies (n = 66) by immunohistochemistry. Dark brown colour indicates positive staining, whereas the cellular structure is visualized by haematoxylin counterstaining. Note the higher number of infiltrating cells in E‐PBC compared to L‐PBC. Magnification ×200

The main morphology changes of PBC were in portal tracts. The liver tissues in early stage showed the damage of bile duct and obvious proliferation of small bile ducts. The granulomas and lymphoid follicles were found in the liver tissues of PBC (Figure 1). There was significant difference between early stage and late stage in the presence of granulomas, lymphoid follicles, damage and proliferation of bile ducts in portal areas (Table 2). Severity of fibrosis was demonstrated in late PBC (Figure 1).

**Table 2:** Histological features of patients with PBC and presence of emperipolesis

	E‐PBC group (n = 39)	L‐PBC group (n = 27)	Statistical value (*χ* ^2^ or *t*)	*P* value
Granulomas	10	0	8.159	.0043
Damaged bile duct	26	3	19.99	<.0001
Bile duct proliferation	27	7	11.98	.0005
Emperipolesis	6.750 ± 1.060	2.625 ± 0.885	2.766	.0127

**Figure 2:**
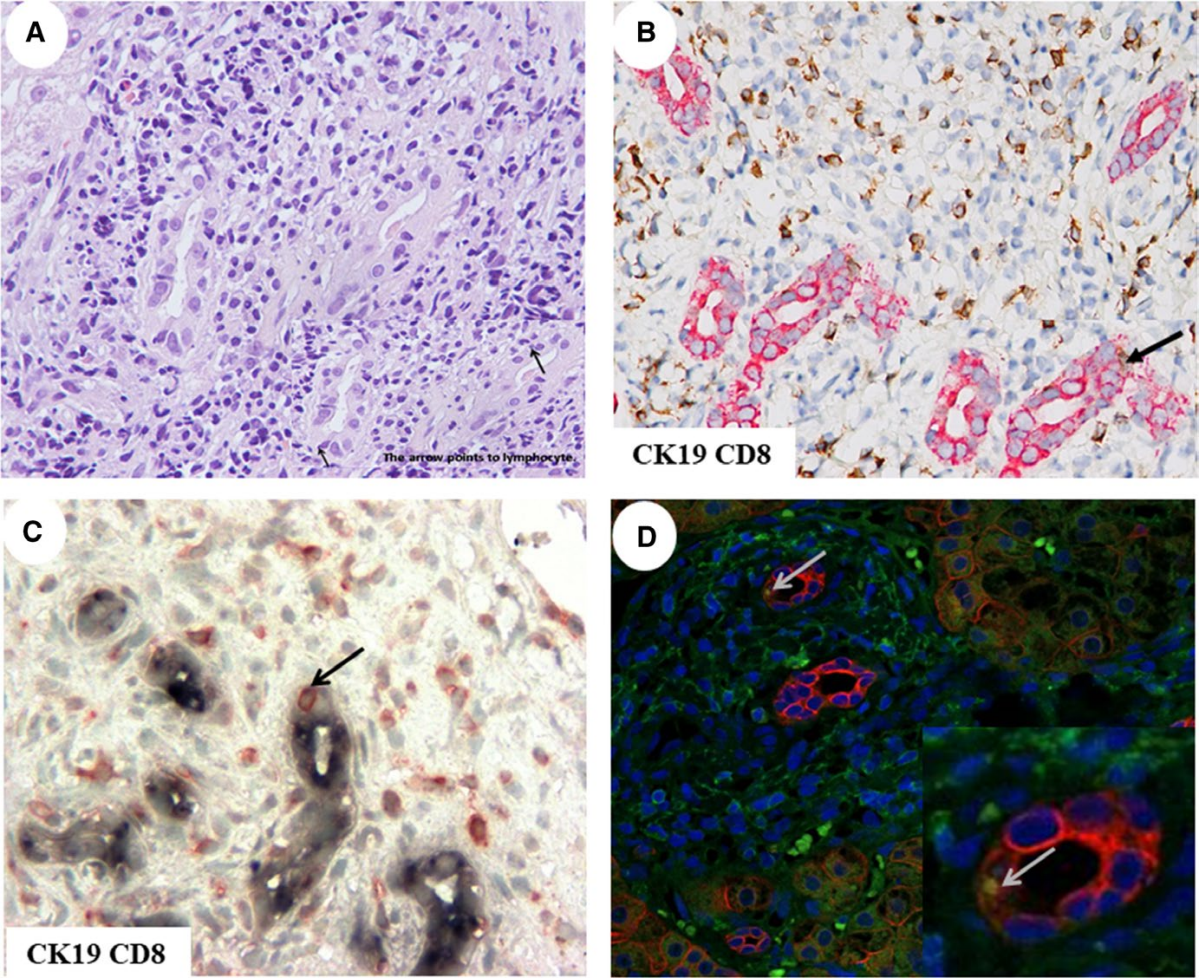
Emperipolesis in liver tissue from PBC patients. A, Representative images of emperipolesis in H&E stained liver sections from patients with PBC. The arrow points at the site of emperipolesis. Magnification ×400. Representative images of IHC and IF double staining of the structures affected by emperipolesis (B‐D). CD8^+^ T cell was the invading cell. B, IHC double staining. BEC was stained blue, and invading cells were stained red. The arrow points at the site of emperipolesis. C, IHC double staining. BEC was stained red, and invading cells were stained brown. The arrow points at the site of emperipolesis. D, IF stained cells. Invading cells were stained green, and BEC was stained red, the invading cells were stained yellow. Magnification ×400

To address whether the presence of emperipolesis was related to bile duct damage in PBC, we evaluated and compared the frequency of emperipolesis between E‐PBC and L‐PBC patients. The emperipolesis, based on entry of lymphocytes into BECs, was characterized by a halo around the nucleus of lymphocytes in H&E‐ stained liver sections (Figure 2). It was frequently located in portal tracts with lymphocytes infiltration (Figure 2). Emperipolesis was observed in 62.1% (41/66) of patients with PBC. The number of emperipolesis in portal area in E‐PBC was significantly higher than in L‐PBC (Table2).

In PBC patients with emperipolesis, there were more severe changes including portal inflammation, lymphoid follicles, damage and proliferation of bile ducts. PBC patients without emperipolesis also had more advanced fibrosis than those with emperipolesis.

### 3.3 Inflammatory cells infiltration around the injured bile ducts and Emperipolesis

In portal area of the liver tissue from patients with PBC, CD3^+^, CD4^+^, CD8^+^ and CD20^+^ positive cell number increased (Table 3; Figure 2). The numbers of CD3^+^, CD4^+^ and CD8^+^ cells around the damaged bile duct in the early stage were more than that in the late stage. The difference was statistically significant (*P* < .01, *P* < .05, *P* < .01). Compared with E‐PBC, the number of infiltrated lymphocytes in L‐PBC decreased, especially CD3^+^ and CD8^+^ positive cells. There was significant difference between two groups (all *P* < .05).

**Table 3:** The number of positive cells in different groups (mean ± SD)

Cell type	E‐PBC group(n = 39)	L‐PBC group (n = 27)	*P* value
CD3	111.28 ± 16.55	75.22 ± 8.47	<.01
CD4	59.50 ± 47.30	43.00 ± 17.60	<.05
CD8	62.71 ± 14.38	43.81 ± 14.40	<.01
CD20	22.80 ± 15.40	16.636 ± 3.396	<.05
TUNEL	17.46 ± 2.58	4.96 ± 3.16	<.01
Ki67/CK19	3.44 ± 1.62	1.44 ± 1.22	<.05

**Figure 3:**
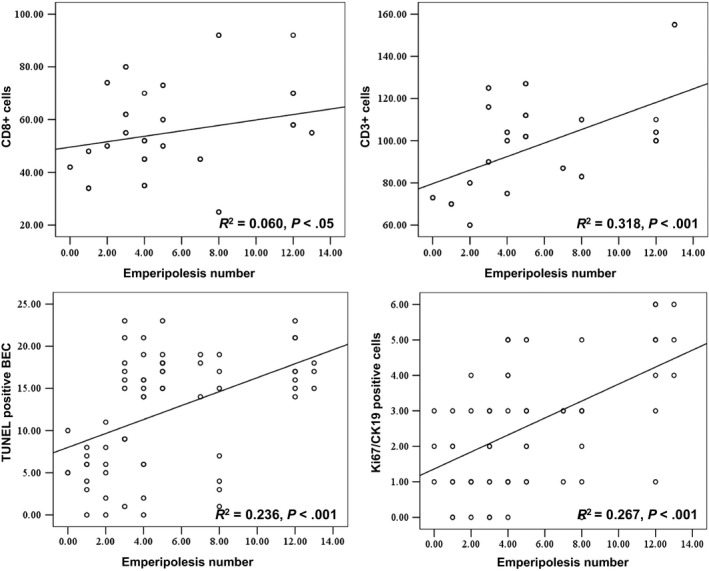
Emperipolesis number and CD8, CD3, TUNEL/CK19 AND Ki67/CK19. The number of emperipolesis per portal tract is related to the number of CD8, CD3, TUNEL/CK19 and Ki67/CK19 in portal area

We performed immunohistochemistry double labelling staining for CD3, CD4 and CD8 T cells, CD20 B cells, and the biliary epithelial cells marker CK19 in the liver sections which showed emperipolesis in H&E stained slides from PBC patients. BEC was stained red, and invading cells were stained brown in IHC (Figure 2B). BEC was stained blue, and invading cells were stained red in IHC (Figure 2C). CD8^+^ positive cells were stained green, and BEC was stained red, and the invading cells were stained yellow in IF (Figure 2D). Contact between CD8 T cells and CK19 was frequently detected, while entry of CD8 T cells into BECs was occasionally observed (Figure 2). The entry of CD4 and CD20 cells into BECs was not detected. CD3 and CD8 T cells correlated with emperipolesis process (*R* ^2^ = 0.318, *P* < .001; *R* ^2^ = 0.060, *P* < .05; Figure 3).

### 3.4 Emperipolesis correlated with apoptosis and proliferation of BECs

**Figure 4:**
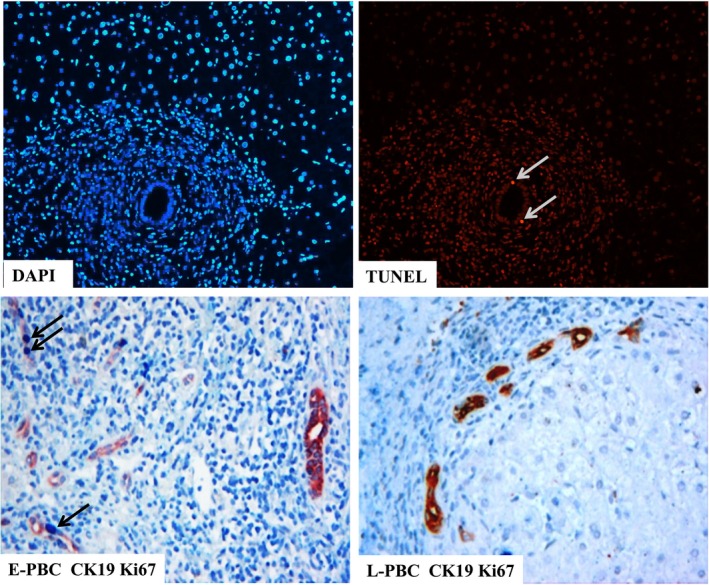
Apoptosis and proliferation of BEC. Representative images of TUNEL staining (red) are displayed, with DAPI indicating nuclear staining of the cells (blue). Proliferation of BEC was shown by IHC double staining with CK19 and Ki67. BEC was stained red, and Ki67‐positive cells were stained blue

To illustrate the consequence of emperipolesis of CD8 T cells, we observed the expression of apoptosis using the Terminal Deoxynucleotidy Transferase (TdT)–mediated dUTP nick‐end labelling (TUNEL) assay during emperipolesis. Proliferation of BECs was evaluated with double staining of CK19 and Ki67. We chose liver sections in which emperipolesis numbers exceeded 5 by H&E staining to increase the positive detection rate. TUNEL‐positive BECs were stained red (Figure 4). Ki67 and CK19 positive cells were stained blue and red, respectively (Figure 4). The number of Ki67 and CK19 double positive bile duct was counted. The results showed that TUNEL‐positive BEC, CK19 and Ki67 double positive cells in the E‐PBC group were more than those in the L‐PBC group. There was significant difference between the two groups (*P* < .01, *P* < .05). TUNEL‐positive BEC were correlated with emperipolesis (*R* ^2^ = 0.236, *P* < .001; Figure 3). Double staining cells of CK19 and Ki67 were correlated with emperipolesis (*R* ^2^ = 0.267, *P* < .001; Figure 3). In early stage of PBC, the entry of CD8^+^ T cells into BECs was observed and correlated with TUNEL and Ki67‐positive BECs but was not found in late stage.

## 4 DISCUSSION

In our study, emperipolesis of lymphocytes in BECs was more likely to be observed in E‐PBC with lymphoid follicles, damage and proliferation of bile ducts, and inflammatory cell infiltration. Emperipolesis is associated with inflammation and reduced in the repair of fibrosis. These features may be closely related to injuries of bile duct and may eventually be followed by the development of fibrosis and cirrhosis in PBC. Our previous study had shown emperipolesis was accompanied with severe inflammation of CHB including interface hepatitis, confluent necrosis, focal lytic necrosis and portal inflammation.^7^ So, emperipolesis may be one of the initiating factors of the disease.

Lymphocytic infiltration around interlobular bile duct in liver portal area is one of the histological features of PBC. Our results showed that most of the infiltrating lymphocytes were T lymphocytes which were consistent with the findings of Ichik.^9^ In our study, the proportion of CD4^+^ cell in the portal area is relatively high in E‐PBC. CD4^+^ lymphocytes develop into Th1 and Th2 cell subsets, which can generate the respective cytokines. Th1 cells are the main cells that mediate cellular immune response,^10^ which is consistent with the distribution of CD8^+^ around the small bile duct. We speculate that CD8^+^ cells may kill target cells directly or mediate apoptosis, which leads to the damage of small bile duct. Therefore, cellular immune injury may be one of the important mechanisms of PBC. T cells immune function disorder may play an important role in the pathogenesis of PBC.^11^, ^12^

Our study showed that CD20^+^ lymphocytes increased significantly in PBC. We speculate that humoural immunity may also involve in the inflammatory reaction of PBC. The study showed BECs invasion by T cells might be promoted by the CD5‐B cell population in liver tissues of PBC patients.^13^ Ballot and others’ studies also support our results.^14^ They found a significant increase in peripheral blood B lymphocytes, resulting in immune disorders and the occurrence of autoimmune reactions.

We observed the close contact of T lymphocytes and epithelial cells in the early stage of PBC. Further, our results showed CD8^+^ T cells were around the small injury bile duct by immunohistochemical double labelling staining. Cytotoxic T lymphocytes mediated the damage of BECs by two pathways 11, 12: (1) Binding of Fas and FasL resulted in the apoptosis of target cells. (2) TCR of CTL binding with MHC antigen on the target cells, discharge of perforin and granular enzyme led to the apoptosis of target cells. Harada et al suggested that apoptosis of BECs in the liver of PBC might be triggered by the interaction of Fas ligand on the surrounding inflammatory cells and the Fas receptor on the BEC.^4^

Cell‐in‐cell phenomena have gained more attention over the recent years after being ignored for almost a century. Their biological mechanisms and pathogenic roles are starting to emerge. Some findings suggested that lymphocytes may use the alternative cell‐in‐cell phenomenon to kill target tumour cells.^15^, ^16^ Alternatively, tumour cells may destroy invading lymphocytes as a mechanism to escape immune surveillance.^17^ The consequences of emperipolesis were also debatable. We found that CD8 T cells were the major cell type of lymphocytes infiltrating BECs in PBC.

The fate of inner cells in cell‐in‐cell structures had been reported to be partially dependent on the properties of the invading cells.^18^, ^19^ We found emperipolesis of CD8 T cells appeared to be related with apoptosis and proliferation of BECs. In addition, the up‐regulation of WAF1 and p53 related to biliary apoptosis is found in cholangiocytes of PBC,^4^ and significantly greater apoptosis has been demonstrated in cholangiocytes of PBC patients than of other chronic cholestatic diseases, even when controlling to similar degrees of inflammation.^4^, ^20^ Enhanced apoptosis has been implicated in several autoimmune diseases. Our study suggests that BEC apoptosis and proliferation occur commonly in E‐PBC. In early stage of PBC, the entry of CD8^+^ T cells into BECs was observed and correlated with TUNEL‐ positive BECs but was not found in late stage. In early stage of PBC, the biliary epithelial cells were damaged by apoptosis and proliferation. The injured cells were eliminated, and the cells proliferated in order to replace the apoptotic cells simultaneously.

## 5 CONCLUSION

In conclusion, the main immune cells in liver tissues were CD3+, CD4+, CD8^+^ lymphocytes, and CD8^+^ T cells were predominant around the damaged interlobular bile ducts in early stage of PBC. However, in the late stage of PBC, all the cell number decreased. According to our results, the changes of number, proportion and distribution of immune cells in PBC liver tissues showed the damage of intrahepatic bile ducts was mediated mainly by cell immunologic injury through Th1 way. In early stage of PBC, emperipolesis mediated by CD8^+^ T cells appeared to be relevant to apoptosis of BECs and further led to the injury of interlobular bile ducts.

## CONFLICT OF INTEREST

No conflict of interest claimed.

## AUTHORS' CONTRIBUTIONS

YN and JZ designed the research; SZ, LW, NF, GZ and SL performed the experiments; SZ, WL, LJ, YZ and RW analysed data; YN, SZ and JZ wrote the paper.
